# Unveiling hidden aspects of GPS deployment on wildlife: A multistep and transdisciplinary approach to urban wild boar monitoring

**DOI:** 10.1016/j.mex.2024.102931

**Published:** 2024-08-28

**Authors:** Carole Marin, Laurent Couderchet

**Affiliations:** a Centre National de la Recherche Scientifique (CNRS), Laboratoire Passages CNRS 5319, Maison des Suds, Esplanade des Antilles 33600 Pessac, France; b Université Bordeaux Montaigne, Laboratoire Passages CNRS 5319, Maison des Suds, Esplanade des Antilles 33600 Pessac, France

**Keywords:** GPS tracking, Capture-mark-recapture, Urban wild boar, Transdisciplinary, Technical constraints, Human-related dimensions, GPS tracking and capture-mark-recapture of urban wild boar

## Abstract

Studies of free-ranging wildlife often involve individual tracking by sequentially recording animals’ positions over a continuous and extended period. Automatic, programmable, operational continuously, and user-friendly thanks to the development of intuitive software, GPS (Global Positioning System) enable the acquisition of large quantities of data, day and night, regardless of field and weather conditions, while allowing for levels of spatial and temporal resolution in the location data never before achieved in wildlife tracking studies. However, GPS collars deployment on wild fauna does not directly translate into scientific outcomes. This article delves into the hidden aspects of telemetry programs, offering a reflective account of our transdisciplinary experience (between researchers and wildlife managers) in GPS tracking of urban wild boar. The described protocol and its discussion aim to outline the necessary conditions to benefit from GPS programs. The program first requires a common construction of the protocol, which meets the objectives of each partner. Second, raw data collection and transformation into information involve four steps. Finally, both technical and human-related dimensions are to be anticipated and considered for further analyses.

• Transdisciplinary research requires a common construction of the protocol in line with the research question.
• Technical constraints and negotiations between partners need to be considered.
• Multiple steps are required to leverage the scientific advantages of the monitoring.

## Graphical abstract

**Figure:**
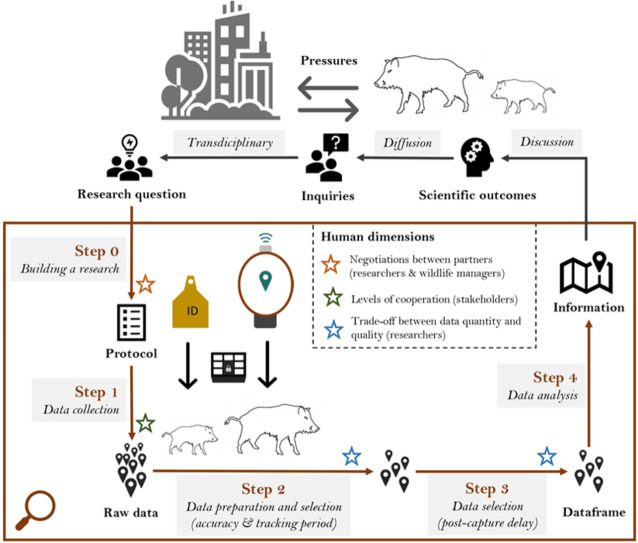

Specifications table

**Table:** 

Subject area:	Environmental Science
More specific subject area:	GPS tracking of Wildlife
Name of your protocol:	GPS tracking and capture-mark-recapture of urban wild boar
Reagents/tools:	Litetrack Iridium 750 PB GPS collars for wild boar
Experimental design:	This protocol article complements a study aiming at better understanding spatiotemporal behavior of urban wild boar, which knowledge is still incomplete. Based on our transdisciplinary experience (between researchers and managers) in telemetry tracking of urban wild boar, we propose a reflective account of essential conditions required to take scientific advantage of GPS deployment on wildlife. Collecting and transforming raw GPS data into usable information is sequential. We break down the critical description of the process into steps: Construction of a research protocol (Step 0); Data collection method (Step 1); Control of biases (Steps 2 and 3) and Data analysis (Step 4). We expose the relevance to: 1) construct the protocol in line with a research question which can meet the objectives of both parties and 2) to be aware of technical limitations and human-related dimensions.
Trial registration:	33–2019–07–23–002 and 33–2020–09–03–004 (French Administration Permits Numbers)
Ethics:	The research complied with the ARRIVE, ASAB/ABS and French National Charter on the Ethics of Animal Experimentation guidelines. It was carried out in accordance with the EU Directive 2010/63/EU for the protection of animals used for scientific purposes, its transposition into French law (French Rural Code, Art R214–87 to R214–137) and the specific environmental legislation (French Environmental Code, Book IV). Legal requirements and ethical guidelines for the care and use of animals were strictly adhered to. Protocols for captures, handling, equipping and releasing free ranging urban boar were approved by French Administration (33–2019–07–23–002 and 33–2020–09–03–004 Permits Numbers) and landowners’ consents were obtained for access to the animals. All procedures involving animals were performed under the supervision of qualified staff. 30 females and 29 males wild boar were captured and ear-tagged, 6 females and 8 adult males were also fitted with GPS collars.
Value of the Protocol:	• The recent democratization of GPS technology has led to a widespread use of GPS tracking devices on wildlife • Operational conditions of telemetry tracking directly impact the scientific outcomes of the program • This article provides a detailed, reflective and critical description of the construction and implementation of a transdisciplinary research protocol involving GPS tracking of large mammals

## Background

Deployed across a wide variety of wild species, GPS (Global Positioning System) technology opens up various study perspectives, including the examination of animal territories (home ranges, structural and functional habitats), spatiotemporal animal behavior (movements, mobility, and activity rhythms), ecological corridors and migration routes, as well as intra- and inter-specific interactions [[1], [2], [3], [4], [5]]. GPS technology has become indispensable in behavioral and movement ecology, to the point of *“shaping the very structure of the discipline of ecology”* [6]. The *“GPS craze”* [7] is reflected in the significant increase in scientific publications related to wildlife telemetry since the early 2000s, the development of collaborative networks for data sharing and exchange, and the emergence of new data analysis methods [[8], [9], [10]]. Widely used in the life sciences, GPS technology is now drawing interest in the social and human sciences as a boundary object to foster dialogue among stakeholders [11], and as a means of narrating coexistence between humans and wildlife through individual animal geographies [12]. Finally, the improvement in wildlife knowledge, allowing for the refinement of management strategies, led to an increasing deployment of GPS technology on various wildlife species by wildlife managers since the 1990s.

In France, enthusiasm for GPS technology has sparked numerous initiatives by naturalist associations and hunting federations for telemetry tracking of wildlife across diverse habitats. While advantages of GPS are undeniable, the widespread adoption of this technique immediately raises questions about animal welfare, the motivations of humans engaged in the program, the technical and logistical constraints, and the future of the collected data. Even though they directly impact the outcomes of the program, the operational conditions of telemetry tracking often remain the hidden aspect of these studies. However, delving into their analysis reveals insights that transcend the mere examination of GPS data points. This article offers a reflective account of our transdisciplinary experience (between researchers and managers) in telemetry tracking of urban wild boar. We review the essential conditions required to take scientific advantage of the system, presented as steps: Construction of a research protocol (Step 0); Data collection method (Step 1); Control of biases (Steps 2 and 3) and Data analysis (Step 4). We discuss the constraints and limitations encountered at each of these steps. The program provides a unique opportunity for participant observation, immersing researchers in the interventions of wildlife managers. Negotiations between partners highlight the complexity of the interaction between two worlds with different objectives. We consider these negotiations an essential part of the research process.

## Description of protocol

### Step 0 – Building a research

Over the past few decades, the democratization of GPS technology has led to a widespread use of GPS tracking devices on animals. While some programs may aim to address scientific and management inquiries, others would have emerged solely because the current technology makes them feasible [7,13]. Our field survey confirmed the trend of wildlife managers orienting studies based on the available technology: drones, thermal cameras, and now GPS. Technology, with its promise of precise and objective animal observations, might even provide answers to poorly framed questions. The technical device is no longer a tool used in a scientific approach; it becomes the scientific method itself. However, telemetry tracking is an expensive and labor-intensive process that involves capturing animals and fitting them with GPS collars. The decision to track animals should come from a cost-benefit analysis, ensuring that the knowledge gained from the study outweighs the negative impact on their welfare [9,14,15]. The development of a research protocol before launching any project is therefore a matter of both scientific rigor and animal experimental ethics, as the two are intertwined.

This study was initiated by the Regional and Departmental Hunting Federations and supported by the French administrative departments responsible for managing wild boar populations. In this local context, management policies face conflicts between humans and urban wild boar, as well as tensions among humans regarding the efficiency of their management, all exacerbated by data deficiencies [16]. This transdisciplinary research aimed to provide new insights into the spatial ecology of wild boar within the urban “landscape of fear” [17], which are essential for enhancing urban wildlife management strategies. The detailed analyses are covered in another article [18] and are not the focus of this contribution. Here, we stress the need to construct the protocol in line with a research question, well before the deployment of GPS collar. At this stage, decisions revolve around:

• Programming the collars (timing and frequency of location measurements, which in turn determine the maximum tracking duration)
• Identifying capture sites, which influence the requests for authorizations
• Determining the characteristics of the GPS-tracked animals (age, sex, weight, which dictate collar size)
• Handling conditions, such as whether to use general anesthesia or not

In our experience, intersectoral collaboration engendered discussions among partners on the three first points.

Previous observations of animal activity rhythms informed the initial research protocol, which envisioned programming collars to record locations every 15 min at night (when animals were expected to move towards sensitive sites) and only twice during the day (when animals were expected to rest under dense vegetation). After joint reflection, it was decided to revise the research approach, moving beyond initial assumptions about animal spatio-temporal behaviour to also investigate daytime movements and habitat use (see Step 4). Additionally, the collaborative preparation of the protocol quickly led the team to consider data loss issues (whether due to receiver non-detection or the deletion of data insufficiently precise; see Step 2), which could result in a lack of daytime location readings. Discussions ultimately led to programming a frequency of position measurements every 30 min regardless of the time of day.

The second point of discussion related to the different urgencies faced by the partners. The program attempted to study urban wild boar, but some arguments presented by the operational partners led to a joint consideration of the capture site selections. The first argument for distancing capture zones from urban areas was based on operational efficiency, suggesting that relocating operations to more rural sites would allow for quicker captures, while the animals would eventually move into urban areas. The following arguments questioned the very objectives of the study, exposing that studying wild boar in rural areas would provide insights into their use of crops and the effects of hunting on their spatial behaviour. While these propositions aligned with the hunting organization's main concerns -compensating for agricultural damage and regulating the species through hunting- it was collectively decided that capture sites would still be located in urban areas, as wild boar encroachment into cities is posing new management challenges.

Finally, there was a discussion about selecting which captured animals would be fitted with GPS collars. Understanding the dispersal of juveniles from urban areas is crucial for managers, and equipping both juveniles and adults would allow for a comparison of their behaviour. However, the GPS-collars are not adjustable and weigh 1 kg. As we collectively decided to prioritize animal welfare, only females weighing at least 45 kg and males weighing at least 50 kg were eventually fitted with GPS collars (see Step 1). In contrast, all captured individuals were ear-tagged and included in a capture-mark-recapture campaign.

From these initial choices stem all downstream project information. In this regard, there is a necessity for clear communication, mutual agreement, and adherence to the agreed-upon protocols, while still meeting the objectives of both parties (fundamental research or operational expectations). This approach can subsequently reduce the need for negotiations between partners.

### Step 1 – Data collection

Marking and telemetry tracking are governed exclusively by the French Environmental Code and, by extension, by environmental ethics (Rural Code, Art R214–88; Ministerial action service order DGAL/SDSPA/N2013–8095). However, capturing, handling, and carrying GPS devices may affect animal welfare [14,19,20]. In wild boar, fatal injuries linked to a stress-induced flight reaction have been recorded [21,22], while hyperthermia, either from stress or weather, and myopathy are particular concerns during capture [23]. Stress can lead to modifications in spatiotemporal behaviour post-release, putting animals at increased risk of predation or road collisions (see Step 3). Finally, there is the inconvenience of carrying the equipment. The challenge of maximizing animal welfare is ethical. It is also scientific, as the physiological, cognitive, emotional and health status of the animal affects the quality of research outcomes [15,24]. However, wild animal welfare would still occupy a largely insufficient place in the construction of experimental protocols [25]. One explanation lies in the lack of understanding of the 3R principle among some wildlife managers involved in monitoring programs ([26]; personal observations). Furthermore, while ethics of experimental practices rely on recognizing the sensitivity of animals, reflecting a welfarist view of animal ethics inspired by the works of Peter Singer [27], researchers and managers involved in tracking programs would adopt environmental ethics [26], which center its values on the populations and ecosystems above the integrity of the individual living being. Finally, the 3Rs rule was initially developed to improve the welfare of laboratory animals, and applying it to free-ranging wildlife can be challenging. Consequently, principles guiding monitoring protocols may sometimes prioritize practical considerations over wild animal welfare ([15]; personal observations).

This study was carried out in Bordeaux Metropolis, in the south-west of France. Urban wild boar were captured at 14 different sites by staff from the Gironde departmental Hunting Federation's and Gironde sworn wolf-hunters, from 23 January 2020 to 11 March 2021. Boar presence was assumed based on feedback from stakeholders and confirmed by recording signs of presence and using cameras in the field. The entire procedure gave priority to animal welfare through:

• Captures conducted by operators with extensive experience in capturing large wild mammals
• Supervision by a veterinarian with official certification in the design of research projects involving free-ranging wild animals
• Particular attention to Refinement methods

First, the risk/benefit assessment for the animals and public health concerns led the team to decide not to anaesthetize urban boar (see **Appendix 1**). Each captured wild boar was transferred to a holding cage specifically designed for the needs of the study and then manually handled. Once tagged, animals were released immediately in an alert state. While vigilant handling eliminates risks during induction, narcosis, and recovery, it does not allow for complete immobilization, which is necessary for reliably estimating age by examining dental formula [28]. Therefore, age was determined on the basis of morphological criteria (development of the mammary glands in females, size of the tusks in males, weight, height and conformation). Captured boar were classified into the three age categories classically distinguished for the species: juveniles (under one year old), subadults (one to two years old) and adults (over two years old) [29,30]. Another key issue was the choice of a weight threshold for selecting animals to be fitted from those captured. As fluctuations in weight during tracking period may influence the impact of the equipment on behavior, physical condition and well-being, animals were monitored regularly throughout the study. A detailed description of the entire procedure can be found in Supplementary Information (**Appendix 1**).

Thirty females and twenty-nine males were captured. Two-thirds of them were less than one year old and 83 % weighed <50 kg. This age-related capture bias is a phenomenon well known to trapping practitioners and reported in the literature [31,32]. According to field experts, it would reflect both the local population's demographics and the greater caution of older individuals. Each captured wild boar was marked with an ear tag with a unique identification number. Tags returned by hunters or authorities represent the recapture data. Date, time, location and circumstances of the recapture (hunting, administrative destruction or road collision) were reported and age, general condition and physiological state of the animal were assessed. Six adult females and eight adult males were also fitted with Litetrack Iridium 750 PB GPS collars from Lotek.

In our experience, human-related dimensions to be considered for data collection were related to stakeholders. Most of them saw the research project as an extension of Institutional Hunting's management and political agendas, and the academic contribution was not seen as impartial. From a strong desire to participate to withholding field information, attitudes reflected a spectrum of relationships with the hunting institution, while introducing bias mostly into the capture-mark-recapture campaign. We argue that a clearer presentation of the link between the program and the academic world could have led to more effective collaborations.

### Step 2 – Data preparation and selection

From this step, data were formatted using Excel (MO Professional Plus 2019, 1808 version), statistical analyses were carried out using RStudio 2023.09.0 software, spatial processing and map production were carried out using the geographic information system QGIS 3.34.

Transforming raw GPS data into usable information requires successive stages of data preparation, correction and selection, which are essential to increase the reliability of the study. This involves:

• Formatting and correcting the data
• Selecting data based on an assessment of GPS-error
• Selecting GPS-tracked individuals based on a minimum tracking duration

Positions measured by the GPS receiver were transmitted in packets of 12 to the manufacturer's server via the *Iridium* telecommunications satellite network and then made available on an online service. For each datum, date, time, latitude, longitude, altitude and outside temperature were recorded. We provided the characteristics of the tracked animal (name, sex and age class) and the location of the capture. To facilitate analysis, we created a column showing the order in which the observations were made. We corrected the manufacturer's pre-programmed time, and therefore sometimes the date. Indeed, we noticed a systematic difference of one or two hours between the time entered and the time corresponding to our time zone, depending on whether we are in “winter time” or “summer time”.

Under field conditions, various natural physical obstacles (dense vegetation or relief) and artificial obstacles (buildings, signal interference zones) between the collars and the GPS or telecommunication satellites [8,33,34], as well as the behavior and lifestyle of the equipped animals [35,36] are likely to affect the performance of GPS collars. In our study, 14 animals were tracked for a total of 1428 days (mean 102.0 ± 79.5 days). This campaign resulted in 47,361 location records out of an expected 68,692 with pre-programmed devices of 48 locations per day. The Fix Status Rate (FSR) was 65.9 % (± 18.4 sd). From these data, we selected those associated with satisfactory accuracy. Each location is associated with a fix status and a Dilution of Precision (DOP) value, which depend on the number and three-dimensional positioning of satellites used to calculate the position of the GPS receiver. The DOP value is usually used to filter the data: the lower it is, the more accurate the location is considered. The manufacturer fills in three categories of data quality: “ideal” when the DOP is <1, “good to moderate” when the DOP is between 1 and 2, and finally, “not very accurate” when the DOP is greater than 3 [37]. However, selecting data on the basis of DOP values categories is not sufficient to guarantee the quality of the monitoring results [[38], [39], [40]]. In order to control for location errors, we carried out performance tests on the collars before their deployment on animals. We determined DOP values and location statuses associated with adequate precision, assessed by measuring distances between the real positions and the recorded locations. We conducted these tests under five different static conditions: collar placed alone or with a second collar in an open space 3 m from the wall of a single-storey house for 24 h; collar placed alone or with another collar in a bush for 24 h (controlled conditions); collar previously worn by an equipped male and left by the animal in a nest with dense vegetation for 6 days (field conditions).

**Fig. 1:**
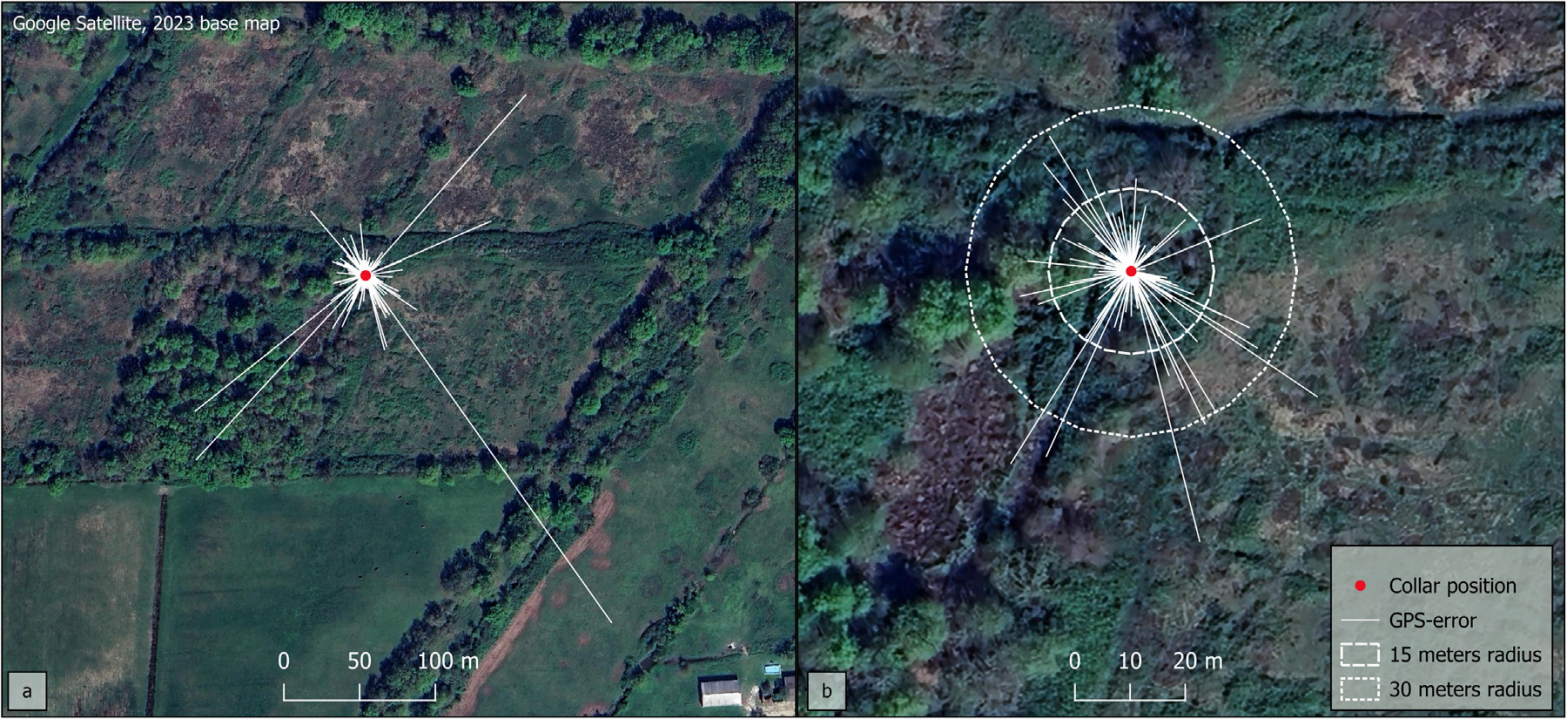
GPS location error under field conditions a) Before data selection b) After data selection.

Under controlled conditions, the average GPS-error was 8.6 m (±23.1 sd) and 91.0 % of location were associated with an error of <15 m. Under static field conditions, the data were slightly less reliable: the average GPS-error was 11.5 m (±24.8 sd) and 81.4 % were associated with an error of <15 m. These measurements are consistent with those from tests conducted under similar conditions, which reported average GPS-errors between 5 and 20 m for different collar models [[41], [42], [43]]. Our tests revealed significant differences between GPS-errors of locations associated with 2D-least square status and those associated with 3D-least square and 4 or more satellites known fix status (F(2761)=76.2, *p* < 2.2e^-16^). The removal of locations associated with 2D least-squares status, as part of the usual methods of location selection [33,44], resulted in a mean GPS-error of 7.6 m (±9.0 sd) under controlled conditions. Using this new dataset, we confirmed that the distance to the actual coordinates tends to increase as the DOP value rises (Pearson's *r* = 0.45, *p* < 2.2e^-16^). We sought a threshold value of DOP by calculating the mean errors and their 95 % confidence intervals for each DOP value. The mean GPS-error of locations with a DOP value ≤ 3.4 were <10 m, and the upper limits of their confidence intervals were <20 m. After data selection, the average GPS-error was 6.6 m (± 6.62 sd) under controlled conditions and 7.0 m (± 7.7 sd) under field conditions. Under controlled conditions, 93.9 % and 98.8 % of GPS data were associated with an error of <15 m and 30 m, respectively. Under field conditions reflecting the resting periods of wild boar, 89.9 % and 97.6 % of GPS data were associated with an error of <15 m and 30 m, respectively (Fig. 1). Data selection improved the accuracy during our tests; it should enhance the quality of the results of GPS tracking. However, this process required removing 25.8 % of the locations collected after deploying the collars.

**Fig. 2:**
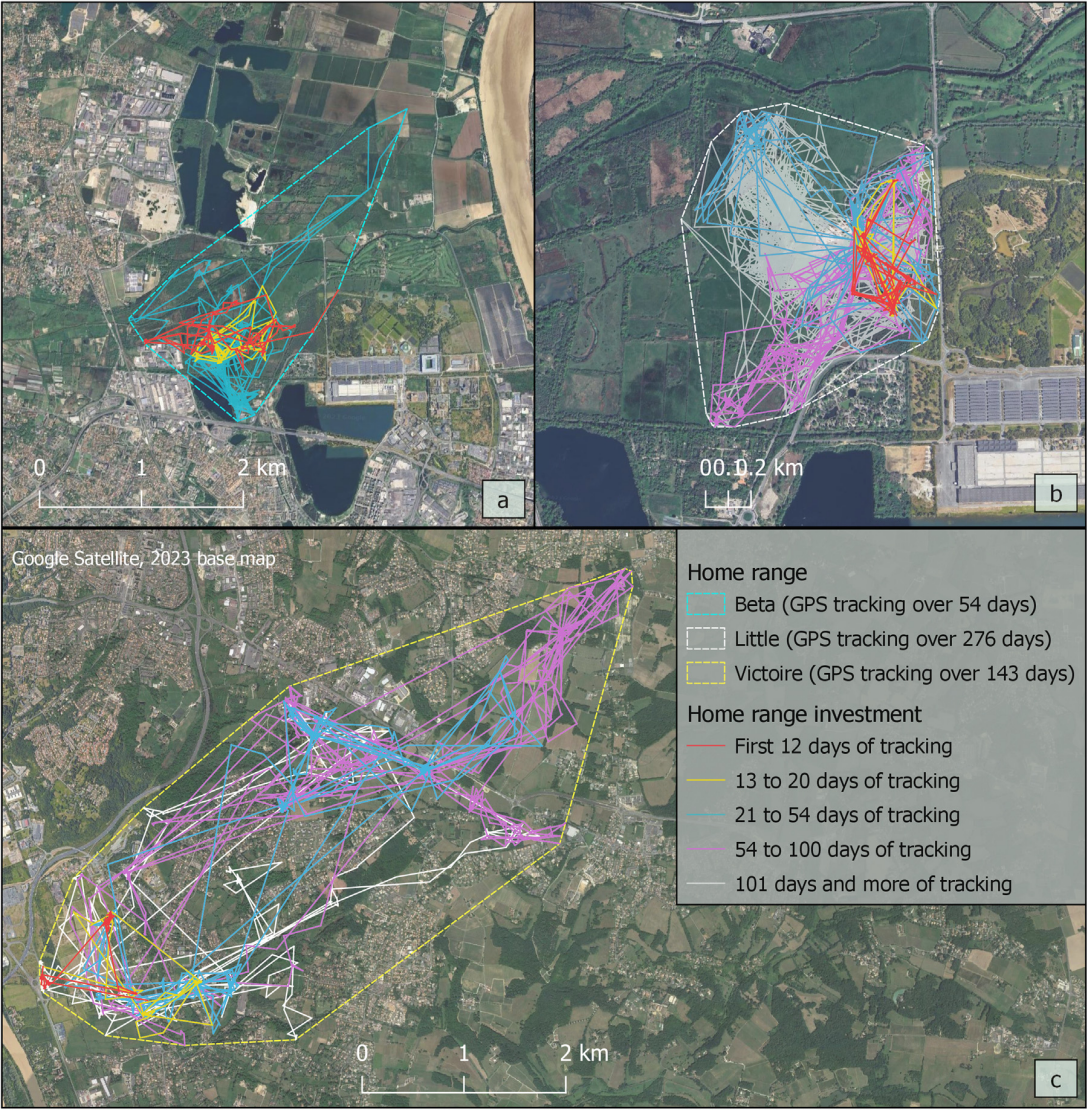
Movements and areas explored in the home ranges as the tracking progressed: a) The individual Beta, an adult male captured in Bordeaux Flower Park, extended his home range beyond 20 days of monitoring. After 54 days, he lost his collar in Bruges National Nature Reserve, and it was removed by reserve staff 5 days later, a few dozen meters away; b) The individual Little, an adult female living in the same area as Beta, remained in the urban forest where she was captured during the first 20 days of monitoring. She then visited the meadows at the north-western edge of the forest until the 54th day, and moved to the south-western edge of the forest between the 55th and 100th day, after which her home range ceased expanding; c) The individual Victoire, an adult female captured in a suburban commune south of Coteaux Park, stayed within a small area around the capture site for the first 20 days before extending her home range to the northeast until the 54th day. Between the 55th and 100th days, she ventured further northeast and southeast, after which her home range extended only slightly southward.

Another key issue was determining the minimum tracking time needed for an individual to fully explore the environments it typically uses. The literature suggests various useful methods to achieve this, such as statistical correlation between tracking duration and home range size [45], incremental accumulation of wild boar home range sizes based on the number of GPS locations collected [46], or testing the range residency assumption implemented in a R package [47], specifically by analyzing the stability of the home range over the tracking duration using variograms. In our study, we combined a statistical analysis and a more empirical method to analyze the relationship between tracking duration and home range size. We first constructed home ranges for each of the 14 boar using the minimum convex polygon (MCP 100 %) method. Statistical treatments showed a trend effect of tracking duration on home ranges area (Spearman's *r* = 0.52, *n* = 14, *P* = 0.06) and a lack of significant influence beyond 12 days of monitoring (Spearman's *r* = 0.38, *n* = 12, *p* = 0.23). We supplemented these statistical analyses by observing home ranges as the monitoring progressed. We estimated movements based on the shortest distance between two consecutive locations. Three examples of these observations are provided in Fig. 2. We finally chose the minimum threshold of 54 days of monitoring, which permitted to include in the subsequent analyses two individuals tracked for <100 days. We therefore selected 10 individuals to be monitored for a total of 1381 days.

**Fig. 3:**
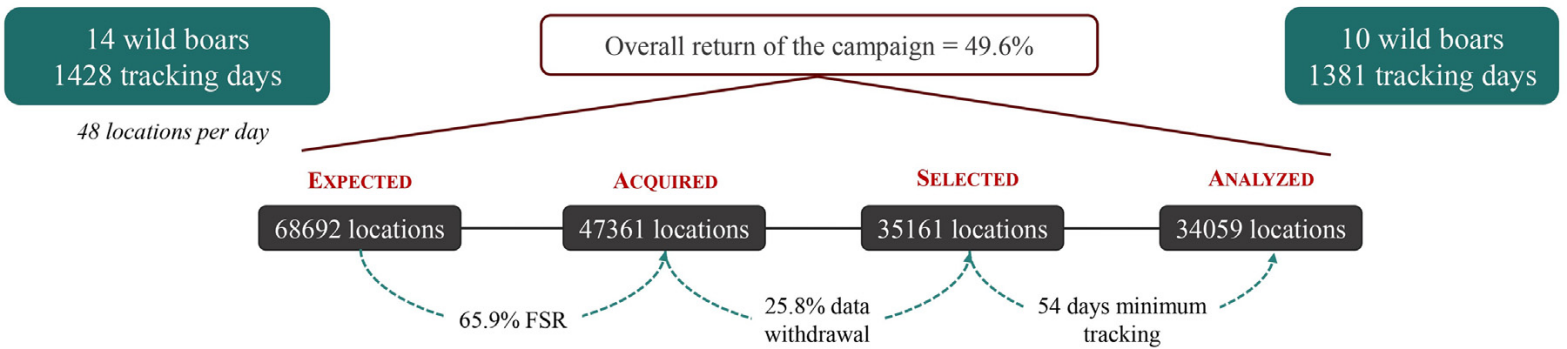
Fix status rate and successive stages of data selection result in overall return of <50 %.

After importing the data into GIS software, we found a few outlying points (isolated and far from the point cloud); their number was anecdotal. In the end, 34,059 locations were analyzed, corresponding to an overall return on the monitoring campaign of 49.6 % (Fig. 3).

### Step 3 – Considering capture effect

Considering a potential behavioural response to the stress caused by capture, handling, and initial outfitting during the early stages of tracking emerges as the final essential prerequisite for studying spatial behaviour using GPS data. Capture is likely one of the most stressful events in the life of a wild animal. Capture-induced stress can lead to avoidance of sources of human disturbance in the early stages following release [5,48], induce a reduction in activity and movements [48,49], or conversely, cause an initial increase in movements following capture [50], both potentially leading to increased vulnerability to predators and accidents.

We explored the capture effect by analysing the mobility and activity of animals fitted with telemetry devices during their 50 first days of monitoring. This duration would exceed the duration of capture effects, which have been evaluated in the literature as 10 days for wild boar [51] and 10 days to one month for roe deer [52], while allowing to include the 10 wild boar selected for the study. Based on the literature and the observation of animals’ behaviour during releases, we anticipated significant movement immediately after release (as the animal would quickly distance itself from the capture site), followed by a phase of reduced activity corresponding to both a state of stress and an adaptation period to carrying the telemetry equipment. To achieve this objective, we:

• Constructed variables to be predicted: “Daily Distance Travelled” (DDT, metres); “Speed of movement” (Speed, metres/30 min) and “Daily activity rate” (DAR,%)
• Calculated their mean values and performed Generalized Additive Mixed Models (GAMM)
• Constructed or simply included in these models several explanatory variables: post-capture periods, season, sex, monitoring zone (see Step 4), outdoor temperature ( °C) and time of day
• Selected and analysed final models

A detailed description of this study can be found in **Appendix 2**.

**Fig. 4:**
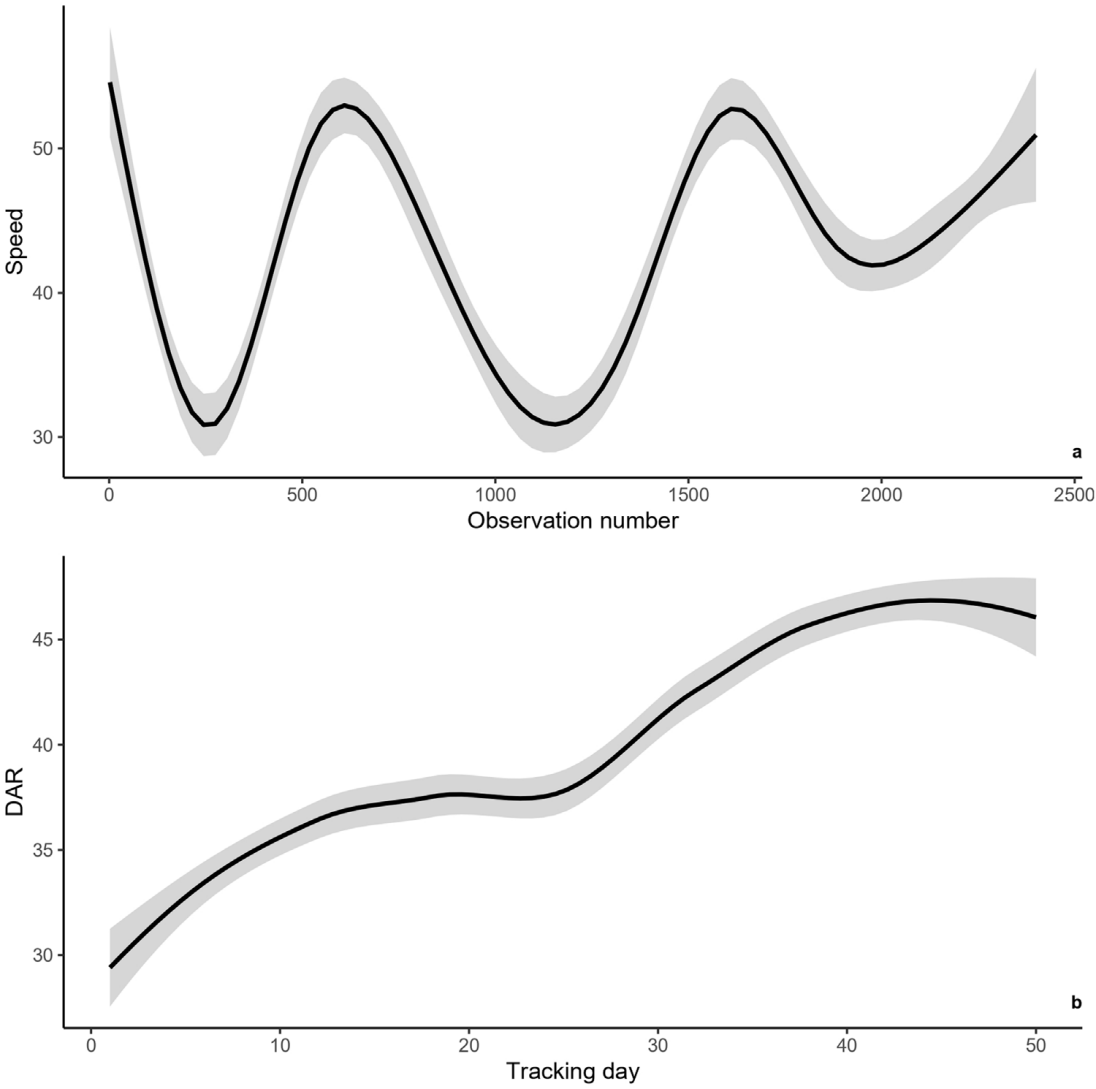
Values of Speed of movements and DAR during the first 50 days of monitoring, predicted by the best GAMM: a) Effect of the post-capture delay on Speed; b) Effect of the post-capture delay on DAR.

Comparison between the mean values of Speed, DDT and DAR calculated over the first 50 days of tracking with those obtained from the entire tracking durations suggested a reduction in mobility and activity during the initial days of monitoring. Indeed, wild boar travelled an average of 1760.7 m per day, with a mean movement speed of 42.8 m per 30 min and an average DAR of 40.1 %, compared to 1974.0 metres per day, 47.0 m per 30 min and 40.1 % for the entire monitoring period, respectively. The results from the GAMMs partially confirmed the hypothesis of a capture effect on the mobility and activity of wild boar in the initial period following their release. When included in the models, post-capture delay had no significant effect on DDT, and the best model did not include this predictive variable. The speed fluctuated over time with no clear pattern (Fig. 4a), and the significant effect of post-capture delay seemed to be related to adjustments of mobilities according to environmental conditions. However, we found a significant effect of post-capture delay on DAR. The main result is that wild boar's activity is reduced during the first month of tracking. Activity levels were low during the first few days, increased over about 12 days, stabilized between the 15th and 25th days post-capture, and then increased again to a higher level beyond the 40 th day of monitoring (Fig. 4b). Detailed results are presented in **Appendix 2**.

These results contrast with those reported in the literature [51], which show a clear effect of capture on both mobility and activity of wild boar during the first 10 days of tracking. These differences could be due to the method of animal immobilization: general anaesthesia *versus* manual restraint using a custom-made holding cage followed by rapid on-site release (see Step 1). While manual restraint avoids the immediate and delayed effects of anaesthesia (see **Appendix 1**), our results suggest that it may induce a longer-lasting reduction in wild boar activity than described in the literature. However, these results should be interpreted with caution. First, our sample includes only 10 animals. Additionally, we lack data on the mobility and activity of these individuals before they were equipped. Furthermore, the discomfort caused by carrying the equipment could add to the stress effects related to capture and restraint.

In order to control biases due to abnormal behaviour after release, authors often choose to omit the first 24 h [2], the first 2 days [46], or the first 3 days [53] of tracking from their spatiotemporal behaviour analyses. We chose to keep the GPS data of all 10 wild boar for the entirety of their tracking period.

### Step 4 – Data analysis

This study aimed to observe the spatiotemporal behaviour of urban wild boar, for which knowledge remains incomplete (see Step 0). Following the selection of GPS data and prior to applying statistical treatments, we first:

• Meticulously observed telemetry data through mapping and graphing
• Constructed study variables based on a comprehensive consideration of the study's objectives
• Constructed environmental variables
• Made thoughtful choices regarding the landcover databases

**Fig. 5:**
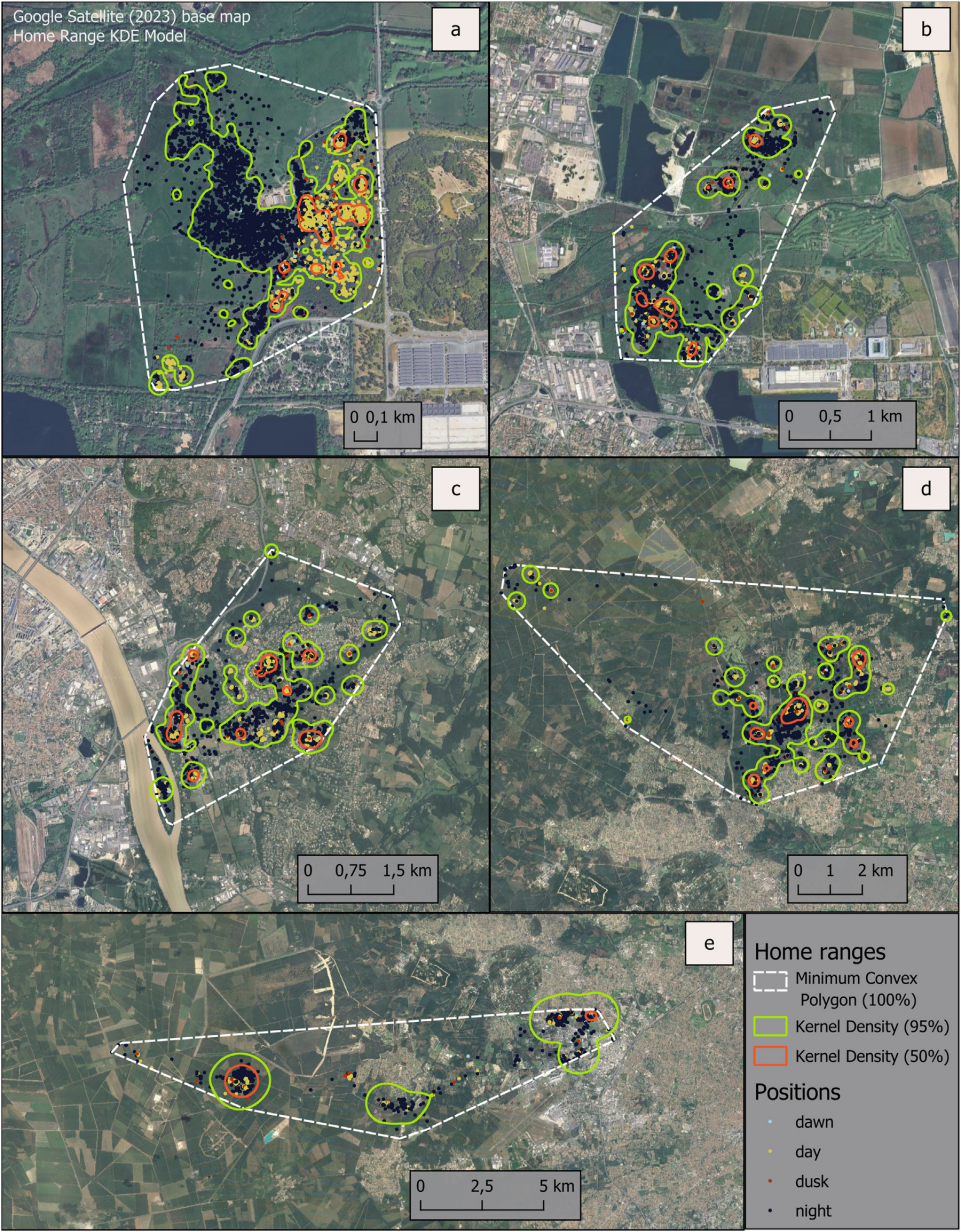
Five telemetry monitoring showing positions by the phase of the day and home ranges: a) The individual Little, adult female captured inside the actual protected perimeter of the Bruges urban National Nature Reserve and tracked for 276 days; b) The individual Night, adult male tracked for 162 days in the same zone than Little; c) The individual Sacoche, adult male tracked for 175 days in a residential area adjacent to the ring road, on the right bank of Garonne River; d) The individual Sunday, adult male caught in the suburban commune of Blanquefort and tracked for 157 days; e) The individual Pangolin, adult male tracked for 143 days in a periurban area located at the north-west of Bordeaux Metropolis. MCP method (100 %) considers all locations, even those corresponding to occasional outings; KDE method (95 %) includes areas necessary for survival; KDE method (50 %) includes the main areas of daytime resting.

In the initial phase, the focus was on observing the distribution of points by the phase of the day, as well as the location, size, and composition of home ranges. We constructed home ranges using the Minimum Convex Polygon (MCP 100 %) and the Kernel Density (KDE 95 %; KDE 50 %) methods. MCP method considers all locations, including peripheral ones which reflect the animal's exploratory behaviour rather than exploitation of the environment or movements necessary for survival and reproduction [54,55]. In addition, polygons include areas that the animal does not frequent and, in some cases, cannot reach [56]. However, the MCP method allows for comparison with the existing literature, as it is widely used to study the spatial ecology of wild boar, sometimes as a complement to other methods. It also allows for assessing the extent of the “explored area” and to study the rejection of the structural habitats that make up the animals’ immediate living space. In contrast, KDE methods are based on delineating zones of equal probability of animal presence. Isopleths with values higher than 50 % and up to 95 % are typically used to delineate home ranges, excluding locations on the periphery of the point clouds [57,58]. Visualisation of location distribution (Fig. 5) suggests three phenomena. First, urban boar are present within the urban fabric and their home ranges are small, except for the most peripheral ones. Second, wild boar appear to forage during the night, with clustered daytime locations and more dispersed night-time locations. Third, they make intensive use of habitats with dense vegetation, especially during the day, while meadows are more frequently used at night. Further analysis may confirm these preliminary observations [18]. To this end, there is a need to carefully considering the construction of variables.

A variety of treatments can be applied to the collected GPS data. What was crucial for managers was to consider differential habitat use during the day and night and to study the factors that influence proximity to urban infrastructure (such as roads and buildings) and distance from forest edges. We have therefore designed a study to investigate these aspects, focusing on home ranges and dispersal of fitted and tagged urban boar, habitat use, risk-taking behaviour, and both inter- and intra-individual variability. The data collected by the collar were rudimentary. Beyond data preparation and selection (see Step 2), their analysis necessitated multiple geomatics treatments to construct study variables [18] (see Step 3 for some examples).

Another important point was the construction of environmental variables. Some animals lived in areas with similar environmental characteristics, even though they were captured at different sites. Several sites were grouped under a categorical variable "monitoring zone", based on two factors: landscape artificialisation and human disturbance within the home ranges (MCP 100 %). We used 6 and 3 criteria to construct the Artificialisation Index (AI) and the Human Disturbance Index (DI), respectively. These criteria are both quantitative and qualitative. Significance of modalities of the qualitative criteria in relation to the indicators of human disturbance and artificialisation were predetermined. For quantitative criteria, the values of each home range were compared to the averages calculated across the 10 home ranges. These ratios indicated whether the indicators of artificialization and human disturbance were negative (-) or positive (+). The positive and negative indicators were then summed to construct AI and DI. Combining these indexes ultimately allowed us to identify three distinct monitoring zones (Table 1).

**Table 1:** Categorisation of home ranges (HR) into 3 monitoring zones with similar landscape artificialisation and human disturbance.

Environmental characteristics		Landscape artificialisation						Human disturbance		
Criteria	Type	Diversity of land cover	Percentage of forest cover	Forest fragmentation	Density of roads and paths	Density of buildings	Main location	Human frequency	Main method of wild boar control	Hunting effort
	Estimate method	Number of different land cover types based on the most detailed level of nomenclature of the regional land cover database* in HR	Ratio of forest area (deciduous, coniferous, mixed) indicated by the regional land cover database* in HR	Natural habitat fragmentation was assessed by calculating Cross Boundaries Connection Effective Mesh Sizes (CBC-MSIZ) of forest environments in each HR⁎⁎	Roads density *R* = *L* / A (where *L* = total length of roads indicated by the French topographic database⁎⁎⁎ and *A* = surface of HR)	Buildings density *B* = *E* / A (where *E* = surface of buildings indicated by the French topographic database⁎⁎⁎ and *A* = surface of HR)	Location of the average coordinates of the HR: in the central city or suburban municipalities or more peripheral	Field observations of traffic intensity (walkers, joggers, cyclists and motorists) on paths and roads	Combination of field observations and administrative reports⁎⁎⁎⁎ on the main methods used to control boar’ populations	Number of drives organised by local hunting associations or wolf-hunters per year⁎⁎⁎⁎
	Modes or Mean value	22.5 different land cover type	43.1 % of forest cover	476.6 forest CBC-MSIZ (ha)	63.5 m of roads / ha	2 % of buildings cover	Central part; Outskirts	Low, Moderate, High	Trapping; Drives; Mixed (trapping and/or drives and/or stalking)	None, Low to Moderate, High
Translation into (+) or (-) indicators	Estimate: HR value / Mean value or Modes	< 1: -; > 1: +; near 1: Neutral	< 1: +; > 1: -; near 1: Neutral	< 1: +; > 1: -; near 1: Neutral	< 1: -; > 1: +; near 1: Neutral	< 1: -; > 1: +; near 1: Neutral	Central: +; Outskirts: -	High: +; Low: -; Moderate: Neutral	Drives and Mixed: +; Trapping: -	High: +; None: -; Low to Moderate: Neutral
AI and DI	Sum of (+) and (-) indicators	Max 1 + and Min 2 - = Score 1 (low AI) 2 to 3 + and Min 2 - = Score 2 (moderate AI) Min 4 + and Max 1 - = Score 3 (high AI)						Max 1 + and Min 1 - = Score 1 (low DI) 2 + = Score 2 (moderate DI) 3 + = Score 3 (high DI)		
Combination 1 of Indexes	4 individuals	Score 2						Score 1		
	“Jalles” zone	HRs in the densely built-up part of the urban area, characterised by a low human disturbance index and a moderate landscape artificialisation index, despite the fragmentation of the forest environment								
Combination 2 of Indexes	3 individuals	Score 1						Score 2		
	“Periurban” zone	Peripheral HRs, comprising large areas of forest with little fragmentation and characterised by moderate levels of human disturbance despite hunting drives								
	3 individuals	Score 3						Score 3		
Combination 3 of Indexes	“Entre-deux-mers” zone	HRs in the densely built-up part of the urban area, characterised by significant fragmentation of the forest environment and road densities, and high levels of human frequency and hunting drives pressure								

⁎ The Regional vectoral large-scale land cover database for Nouvelle-Aquitaine (2015) is produced by photo-interpretation of aerial orthophotographs. The smallest digitised entity measures 1000 m^2^ for built-up areas and 10,000 m^2^ for the rest of the territory. The nomenclature is based on that of Corine Land Cover. Its most detailed level comprises 58 items.

⁎⁎ MSIZ is a fragmentation indicator, which corresponds to the size that the unfragmented habitat fragments in the home range would have if they all had the same size [59]. We used the QGIS “FragScape plugin” and parameterised the algorithm with the Cross Boundaries Connection function to consider the total surface area of forest fragments even if they were partially contained within a home range.

⁎⁎⁎ The French topographic database (2020) provides a 3D vector description of infrastructures, with a metric precision. Each building and road are counted and only their floor area is considered.

⁎⁎⁎⁎ Data from Prefecture and Hunting Federation of Gironde department for 2020 and 2021.

Finally, the choice of landcover databases was crucial not only for constructing the “monitoring zone” variable, but for the overall analysis. The European Corine Land Cover database is often used to study the spatial ecology of wild boar. However, its spatial resolution of 0.25 km2 is not suitable for a local scale study conducted in very heterogeneous urban landscapes. This database generalises human-made environments such as roads and their edges or discontinuous urban fabrics to include small areas of grassland and urban green spaces that could be functional habitats for wild boar. We used the regional large-scale landcover database for Nouvelle-Aquitaine, which has a much finer spatial resolution. To study specifically distances of locations from buildings and roads, we used the French topographic database (2020). More information is available in Table 1.

## Limitations

GPS technology holds the promise of providing a better understanding of wild animal's behaviour. However, the first key question that users must consider is the quantity and quality of the locations, which depend on three main factors: i) the animals; ii) the ability to access them; iii) the tool itself.

Selection of trapping sites depended on both the presence of wild boar and their accessibility to the research team. The willingness of wildlife managers and landowners to cooperate was crucial to the success of trapping operations and was a key factor in site selection. While urban captures appear as much more challenging than rural ones, the morphology of wild boar make it difficult to hold GPS devices on their necks ([10]; personal observations). According to the manufacturer, the programmed recording frequency would have provided a battery life of 2 years. In practice, however, average tracking times was 102 days (138 days for the 10 selected boar), as most wild boar lost and/or destroyed their collars, sometimes after only a few days of monitoring (Appendix 1). Consequently, urban wild boar were mostly monitored during the autumn and winter seasons, compromising the study of seasonal differences in their spatial ecology.

Furthermore, the Fix Status Rate of deployed GPS collars was much lower than the average FSR of 78 % reported in the literature, calculated based on data from 3000 telemetry devices deployed on 62 terrestrial species [8]. This difference suggests the existence of negative effects of urban infrastructure on collar performance. However, further studies seem necessary before any conclusions can be drawn about the limitations of this tool for monitoring urban wildlife. Indeed, the poor performance of the collars could also be attributed to behaviours such as wallowing and rubbing against tree trunks, which can dirty, damage, or alter the position of the antennas (initially oriented towards the sky) [34,36]. This is supported by the very low FSR of the collar on a female (25.8 %), without detecting any malfunction after its retrieval. As the proportions of data associated with a 2D least squares status and a DOP > 3.4 were higher than those obtained during tests in controlled static conditions, almost 50 % of the expected data has been lost. Considering only locations spaced <3 h apart to study movements led to the removal of even more data. We stress the importance of being mindful of the technical limitations of the GPS tool when designing the protocol. Specifically, the significant loss of expected data may warrant pre-programming a high frequency of position calculations to mitigate biases in analysis.

In addition, we established a minimum threshold of 54 days of monitoring, which is either lower [45] or higher [46] than that reported in similar studies. While our study demonstrates a reduction in activity during the first month post-capture, we ultimately decided to retain the GPS data throughout the entire tracking period. These decisions warrant discussion. Indeed, the entire process, including data selection based on an acceptable level of GPS-error, choice of a minimum tracking duration, and disregarding the capture effect, involves a compromise between the quality and quantity of data to be retained for further analysis. With this in mind, we argue that it is appropriate to strengthen the results, firstly by optimising the capture campaign by tagging animals that are too young to be fitted with collars, and secondly by supplementing the study with indirect monitoring methods such as the use of photographic traps.

The second category of technical constraints pertains to the construction of variables for data analysis, specially the activity of wild boar and the type of landcover.

The GPS collars we used did not have activity measuring devices such as accelerometers or head movement detectors. To study wild boar’ activity, we set a movement speed threshold beyond which the locations were considered as “active”. However, while movement is logically associated with a period of activity, activity is not necessarily associated with movement, especially when feeding, drinking and wallowing. In Poland, while activity and movement speed of wild boar in Krakow were correlated, this relationship disappeared for wild boar in the Bialowieza Forest, which may be due to greater local activity of animals using an unfragmented forest environment where readily available resources are evenly distributed [45]. Although our method may lead to an underestimation of animals’ activity, the daily activity rates calculated from the entire tracking dataset align with those described in the literature [18].

**Fig. 6:**
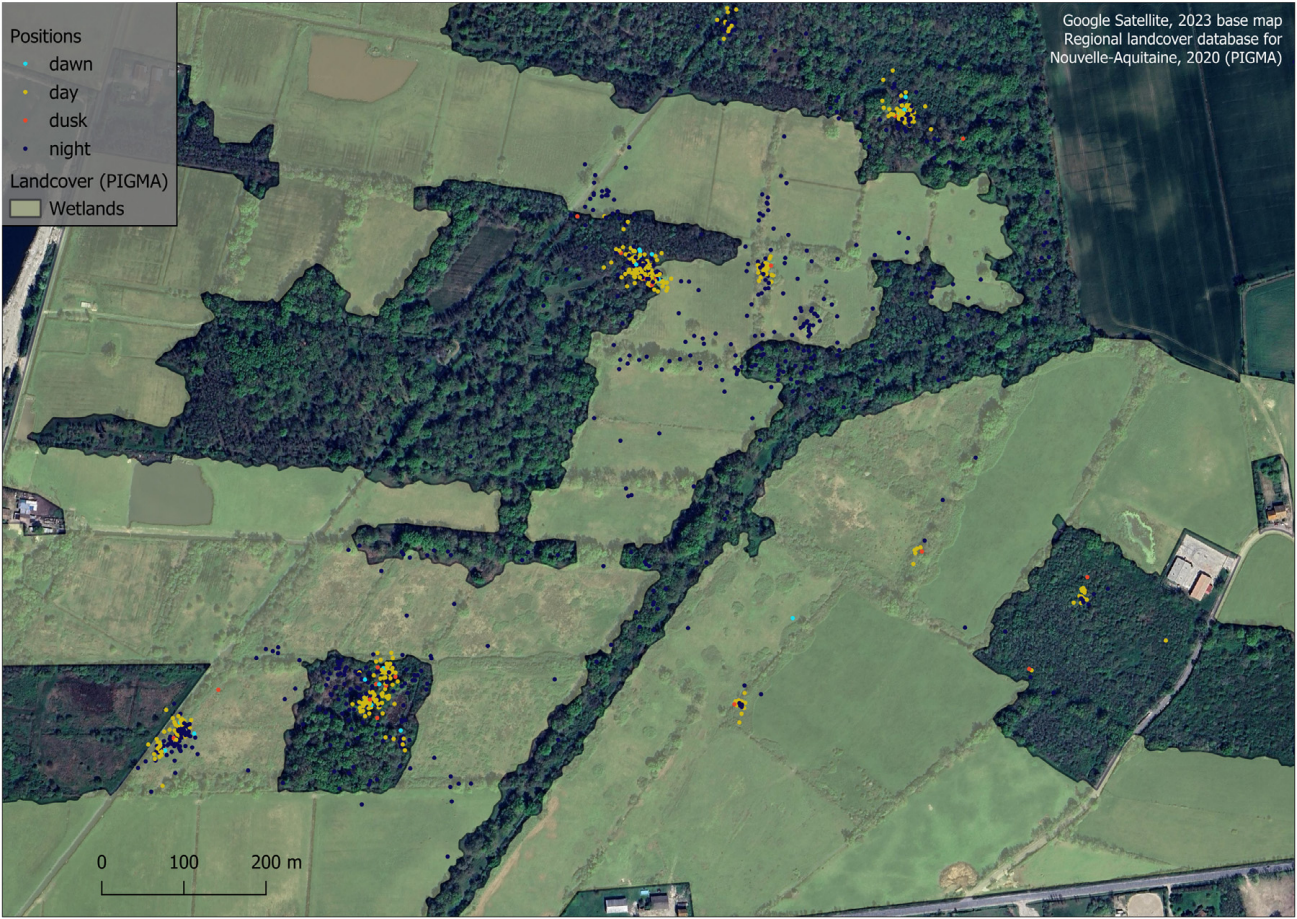
The wooded hedges crossing the wet meadows of the “Jalles” monitoring zone are important wooded areas for the wild boar “Night”, yet undetected by the landcover database.

Finally, the very fine spatial resolution of the regional large-scale landcover database for Nouvelle-Aquitaine allowed for the detection of forest areas used by wild boar, as small as 1.015 hectares. However, this database proved inadequate for detecting treed areas smaller than 1 hectare. For instance, many locations of the wild boar “Night” were found in tree-lined hedgerows of wet meadows. However, these areas were included in the meadow category of the database (Fig. 6). Thus, the use of wooded areas was underestimated, while the use of meadows was overestimated, introducing a bias in the analysis of the diurnal and nocturnal preferences of urban wild boar. Furthermore, the database groups wooded and treed areas into three categories: deciduous forests, coniferous forests, and mixed forests, but differentiating these environments based on their sizes, geometric shapes, and tree density is crucial for studying the preferences of wild boar. This raise the limitations of the spatial and thematic resolutions of general-purpose databases for studying the spatial ecology of animals in detail, echoing the insights of Godard and Capon [60].

## Conclusion

Collecting and transforming raw GPS data into usable information is sequential. Beyond breaking down the critical description of the process into steps, this article aimed to expose the relevance to: 1) construct the protocol in line with a research question which can meet the objectives of both researchers and managers and 2) to be aware of technical limitations and human-related dimensions of such programs.

## CRediT authorship contribution statement

**Carole Marin:** Conceptualization, Methodology, Formal analysis, Investigation, Data curation, Writing – original draft, Writing – review & editing, Project administration, Funding acquisition. **Laurent Couderchet:** Conceptualization, Supervision, Writing – review & editing, Funding acquisition.

## Declaration of competing interest

The authors declare that they have no known competing financial interests or personal relationships that could have appeared to influence the work reported in this paper.

## Data Availability

Data will be made available on request.

## Appendix Supplementary materials

Appendix 1 Detail and illustration of the protocol for capturing, handling, equipping, tagging, and monitoring fitted animals, from setting up the traps to the end of the tracking period. Image, application 1

Appendix 2 Detailed description of the “Capture Effect” study, including the theoretical framework, methodology, and results. Image, application 2
