# Supplementary material for: Unveiling hidden aspects of GPS deployment on wildlife: A multistep and transdisciplinary approach to urban wild boar monitoring
Source: MethodsX. 2024 Aug 28;13:102931. doi: 10.1016/j.mex.2024.102931 (PMC11829123; doi:10.1016/j.mex.2024.102931)
Supplement: Appendix 1 — Detail and illustration of the protocol for capturing, handling, equipping, tagging, and monitoring fitted animals, from setting up the traps to the end of the tracking period. [file mmc1.pdf]

Appendix 1 Capture, handling and monitoring procedures of urban wild boar

Access to boar

Boar presence was assumed based on feedback from various stakeholders and confirmed by recording signs of presence and by using cameras in the field. Trap cages were installed at the sites and put into operation after a few days of acclimatisation.

Selection of trapping sites depended on both the presence of wild boar and their accessibility to the research team. The willingness of wildlife managers and landowners to cooperate was crucial to the success of trapping operations and was a key factor in site selection.

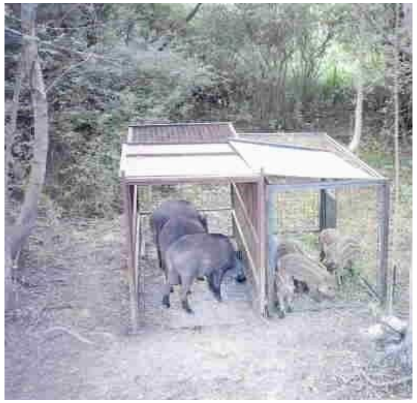

Visit a non-operating cage, Bouliac, 7 July 2020

Capture

Corn kernels placed at the bottom of the cages were used as bait. To reach them, animals broke the fishing line holding the trap door at the entrance, which lowered behind them and the animals were caught. Daily inspection and maintenance of trap cages help to avoid the risk of injury to the animal trapped against the walls.

Cameras equipped with motion detectors, facing the cages, alerted operators when an individual or group approached the cages. Either way, operators visited each trapping site every morning.

Once a capture was confirmed, operators moved quickly to the site to minimise time spent in the cage and to handle the animals at the coolest times of the day.

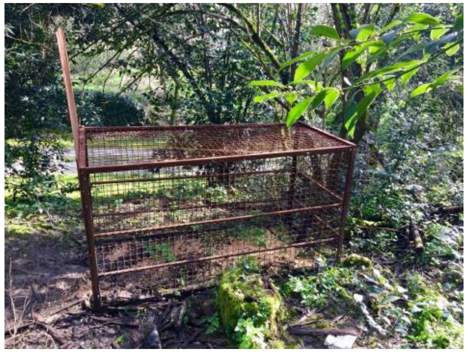

Trap in operation, Floirac, 18 February 2020

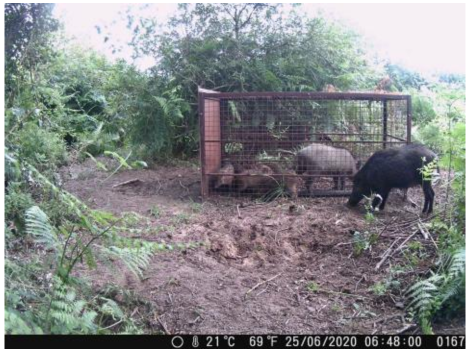

Caught individuals, Bruges, 25 June 2020

Handling & Release

Handling required a minimum of 3 operators. One of them monitored the boar's condition and behaviour and sprayed it with water to avoid the risk of hyperthermia. Amplitude and frequency of respiration and animal's behaviour (alert, agitated or asthenic) were monitored during each manipulation. If an animal showed significant signs of discomfort, it was not restrained for more than 5 minutes before a decision was made to release it.

The risk/benefit assessment for the animals and public health concerns led us to decide not to anaesthetise urban boar we studied*

Each captured wild boar was marked with an ear tag with a unique identification number. Wild boar weighing less than 15 kg were handled outside of the cage. Animals weighing more than 15 kg were transferred to a holding cage located in front of the trap cage entrance. Designed for the needs of the study, this restraint cage was equipped with a sliding wall designed to block the animal being handled**

Dental formula examination is a reliable method for estimating age categories. However, it requires complete immobilisation. As the boar included in this study were not sedated, their age was determined on the basis of morphological criteria (development of the mammary glands in females, size of the tusks in males, weight, height and conformation). Moreover, manual restraint is less comfortable for the handler and adjusting the collar around the animal's neck is more delicate.

The collars are not extendable and the devices weigh 1 kg. The threshold weight for fitting telemetry equipment on wild boar was 45 kg for females and 50 kg for males. The ratio between the weight of the equipment and the weight of the animal was a maximum of 2.2%***

Animal weights and growth rates vary between individuals, seasons, biotopes and physiological stages. Weight fluctuations may influence the impact of the weight of the equipment on their behaviour, fitness and welfare.

Once tagged, animals were released immediately and returned to their groups in a state of alertness.

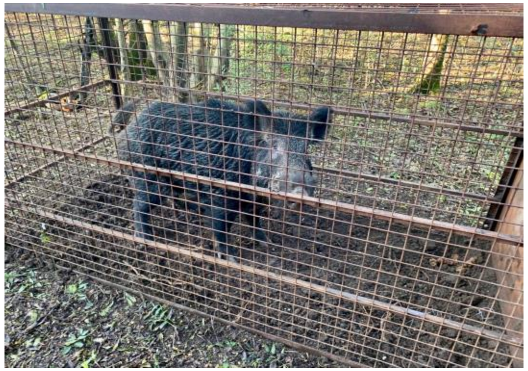

Calm male before transfer to the holding cage, Bouliac, 23 Sept 2020

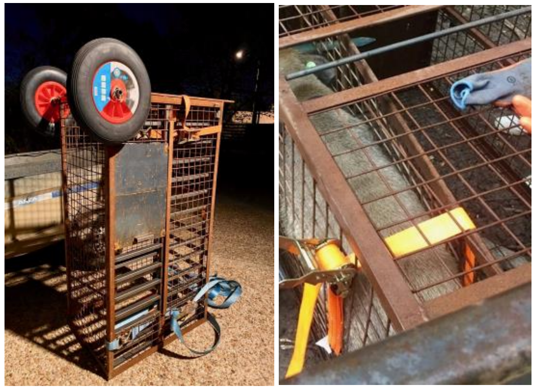

Cage designed for programme requirements, Bordeaux, 28 July 2020

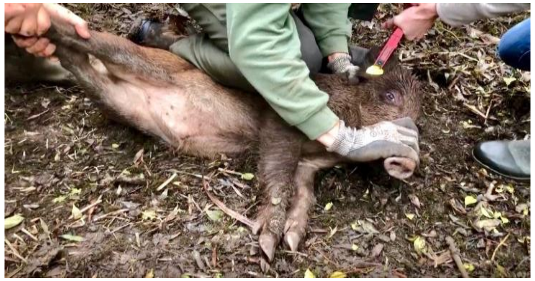

Ear tagging of a young female, Bouliac, 11 October 2020

Monitoring	Boars fitted with GPS collars were primarily monitored indirectly.<br><br>Daily consultation of the interactive map web service allowed for the early detection of unusual spatial behaviour, such as significant deviations from the typical GPS-tracked movement patterns of wild boar. Cameras were placed in areas frequented by the animals to take photographs and videos. If no photos were taken, the boar were decanted to obtain direct visuals. These events were very rare. GPS location from the two hours following the decanting were removed to ensure that our research questions were addressed with minimal interference.<br><br>Animals were then assessed for body condition, locomotion, feeding and social behaviour.	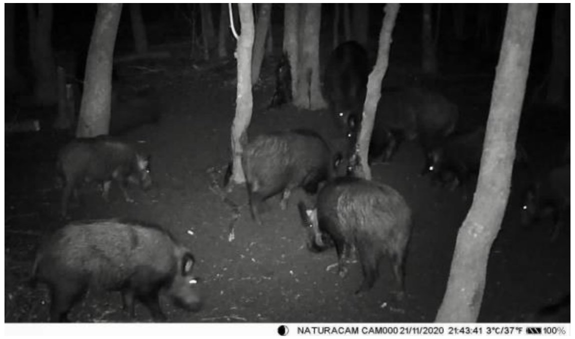 *Victoire 4 months after equipment and its company, Bouliac, 21 November 2020*<br>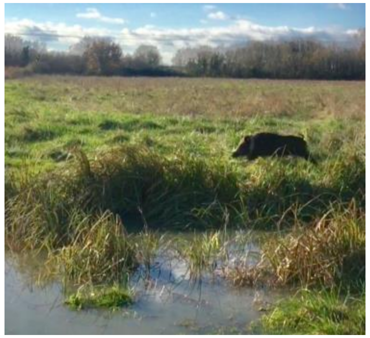 *Sighting of Déjà-vu 6 weeks after equipment, Bouliac, 8 December 2020*
End of the study	9 of 14 wild boar fitted with GPS device lost and/or destroyed their collars, sometimes after only a few days of monitoring. A motion detector alerted operators to the loss of the collar if no movement was detected for 12 consecutive hours. VHF signals were used to locate these collars.<br><br>The tags of 6 of these 9 wild boar were eventually returned: 4 were shot during hunting or administrative drives, 1 was trapped in the National Nature Reserve and removed, 1 was killed in a road collision.<br><br>The others 5 boar fitted with telemetry devices were shot during hunts or administrative removals or shot by a certified wolf hunter regarding endpoints and species-appropriate methods of killing in compliance with legislation and ethical guidelines (Dir 2010/63/EU, Art 6 & Annexe IV).	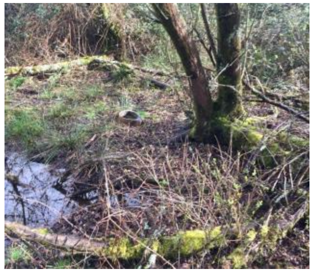 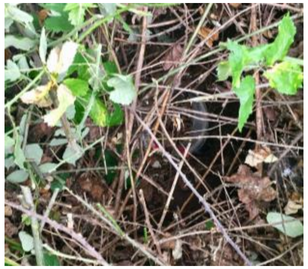<br>*Collars in the environment, Bordeaux, 6 March 2020; Blanquefort, 7 July 2020*<br>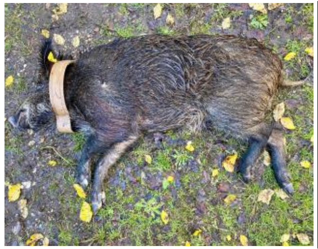<br>GPS locations indicated that this individual was no longer moving, suggesting distress. This observation was confirmed by direct observation of severe lameness.<br>Post-mortem examination revealed a necrotic gunshot wound on the left shoulder, a multiple fracture of the scapula and a perforation of the chest.<br>*Victoire was intentionally shot 5 months after equipment, by a certified wolf hunter for severe gunshot wounds regarding endpoints and species-appropriate methods of killing, 4 December 2020*

*By using manual restraint, we avoided risks during the induction, narcosis and recovery critical phases (Barasona et al. 2013; Fenati et al. 2008), which is all the more important as pre-anaesthetic assessments under field conditions are usually superficial. We also avoided delayed effects of anaesthesia on animals, such as risks of re-narcosis, depression of animal activity and mobility and disruption of circadian rhythms, which regulate cyclic physiological processes (Cheeseman et al. 2012; Poulsen et al. 2018). Vigilant handling significantly reduced the time required to handle the animal: from an estimated 40 minutes with anaesthesia (Brogi et al. 2019) to 10 minutes with our protocol. Chemical restraint also poses health risks associated with the human consumption of animals that have been anaesthetised (Cattet 2003). In Bordeaux Metropolis, wild boar is classified, depending on the area and the time of year, either as a game species or as a nuisance species subject to administrative removal. Although the latter involves destruction by incineration of the carcasses, the venison is shared in both cases (personal observations). When drugs are used, consumption should occur after the appropriate withdrawal times for the prescribed products, to ensure that foodstuffs do not contain residues in quantities exceeding the maximum residue limits of pharmacologically active substances (French Public Health Code, Art L5141-2). However, withdrawal periods for authorised anaesthetic drugs are not given for wild boar.

**The elimination of movement in the handling cage improved operator comfort during handling, speeded up the procedure, prevented the animal from injuring itself on the cage walls and reduced the risk of capture myopathy (Paterson 2007).

***Selection of the animals to be fitted from those captured was a key issue. The weight threshold for fitting telemetry equipment on wild boar was 45 kg for females and 50 kg for males, which is generally reached at one year of age after which the relative monthly weight gain decreases (Klein 1984; Pépin et al. 1987). The risk of strangulation by collars was therefore reduced. The ratio between the weight of the equipment and the weight of the animal at the time of fitting was a maximum of 2.2%, which is lower than the usual recommendations of 3 to 5% (Murray and Fuller 2000; Soulsbury et al. 2020).

Barasona JA, López-Olvera JR, Beltrán-Beck B, Gortázar C, Vicente J (2013) Trap-effectiveness and response to tiletamine-zolazepam and medetomidine anaesthesia in Eurasian wild boar captured with cage and corral traps. BMC Vet Res 9(107):1-11

Brogi R, Brivio F, Bertolucci C, Benazzi M, Luccarini S et al (2019) Capture effects in wild boar: a multifaceted behavioural investigation. Wildl Biol 497:1-10. https://doi.org/10.2981/wlb.00497

Cattet M (2003) A CCWHC Technical Bulletin: Drug Residues in Wild Meat – Addressing A Public Health Concern. Canadian Cooperative Wildlife Health Centre: Newsletters & Publications 46:1-4

Cheeseman JF, Winnebeck EC, Millar CD, Kirkland LS, Sleigh J et al (2012) General anesthesia alters time perception by phase shifting the circadian clock. Proc Natl Acad Sci 109:7061–7066

Fenati M, Monaco A, Guberti V (2008) Efficiency and safety of xylazine and tiletamine/zolazepam to immobilize captured wild boar (Sus scrofa L. 1758): Analysis of field results. Eur J Wildl Res 54:269-274

Klein F (1984) Contribution à l'étude de la croissance du Sanglier (Sus scrofa) par capture et recapture. In: Spitz F and Pépin D (eds) Symposium International sur le Sanglier. Les colloques de l'INRA n°22, pp 55-67

Murray DL, Fuller MR (2000) A Critical Review of the Effects of Marking on the Biology of Vertebrates. In: Boitani L and Fuller TK (eds) Research Techniques in Animal Ecology. Controversies and Consequences. Columbia University Press, New York, pp15-64

Paterson J (2007) Capture Myopathy. In: West G, Heard D and Caulkett N (eds) Zoo Animal &Wildlife Immobilization and Anaesthesia. Blackwell Publishing, Oxford, pp 115-121

Pépin D, Spitz F, Janeau G, Valet G (1987) Dynamics of reproduction and development of weight in the wild boar (Sus scrofa) in south west France. Int J Mamm Biol 52:21-30

Poulsen RC, Warman GR, Sleigh J, Ludin NM, Cheeseman JF (2018) How does general anaesthesia affect the circadian clock? Sleep Med Rev 37: 35–44

Soulsbury C, Gray H, Smith L, Braithwaite V, Cotter S et al (2020) The welfare and ethics of research involving wild animals: A primer. Methods Ecol Evol 11:1164–1181
