# Supplementary material for: Unveiling hidden aspects of GPS deployment on wildlife: A multistep and transdisciplinary approach to urban wild boar monitoring
Source: MethodsX. 2024 Aug 28;13:102931. doi: 10.1016/j.mex.2024.102931 (PMC11829123; doi:10.1016/j.mex.2024.102931)
Supplement: Appendix 2 — Detailed description of the “Capture Effect” study, including the theoretical framework, methodology, and results. [file mmc2.pdf]

Appendix 2 Capture effect study: Theorical framework, variables, analyses and detailed results

Stress is a neurobiological response to external threats (such as predator encounters) or internal events (like injury or a blood pressure drop) that cause pain, discomfort, or survival threats (Koch et al. 2017). Sensory information is processed by the brainstem, leading to the release of catecholamines via the sympathetic nervous system (Ulrich-Lai and Herman 2009). This immediate stress response triggers physiological changes, including increased heart rate and energy mobilization, resulting in the classic "fight, flight, or freeze" reactions (Koch et al. 2017). Delayed stress responses involve the production and secretion of glucocorticoids through the hypothalamic-pituitary-adrenal axis activation (Ulrich-Lai and Herman 2009). In wild boar, this results in elevated cortisol levels (Gentsch et al. 2018) and can influence the animal's spatial behaviour. Capture is a stressful event for wild animals. We explored the capture effect by analysing the mobility and activity of animals fitted with telemetry devices during their 50 first days of monitoring.

We considered three variables to be predicted: "Daily Distance Travelled" (DDT, metres); "Speed of movement" (Speed, metres/30 minutes) and "Daily activity rate" (DAR, %). DDT is the sum of the shortest distances between two consecutive locations on the same day. Speed is the distance between two successive locations divided by the number of 30 minutes periods between them. For this variable, we did not consider locations recorded more than 3 hours apart (32148 locations). Mirroring similar studies (Miettinen et al. 2023; Thurfjell et al. 2014), we used the speed of movement between two consecutive locations as an indicator of the activity, a method validated for two cervid species (Ensing et al. 2014). We chose a speed threshold of 15 metres / 30 minutes. This choice was motivated by the results of the pre-deployment collar performance tests mentioned above: under field conditions and after selecting the data, almost 90% of the locations were actually recorded within 15 metres of the site (see Step 2 in the manuscript). We therefore constructed a binary activity variable, with a value of 1 (for "active") attributed to locations characterised by a movement speed above 15 meters / 30 minutes; and a value of 0 (for "inactive") attributed to those characterised by a speed below this threshold. DAR is equal to the average of the values of P(act) over the same day, multiplied by 100.

We first calculated the mean values of Speed, DDT and DAR for comparison with those obtained from the entire tracking periods. Subsequently, we used generalised additive mixed models (GAMM) for each variable to be predicted. The behavioural plasticity of the species leads to non-linear responses to the various predictor factors, which can be analysed using GAMMs. The identity of the animal can also be included as a random factor in these models. We used "mgcv" R package to build GAMMs and checked their performance using the "gam.check" function.

We included in these models several categorical and continuous variables that could modulate movements and activity: post-capture periods, season, sex, monitoring zone (see Step 4 in the manuscript), outdoor temperature (°C) and time of day. To study movement speed, we used the observation number as the post-capture delay variable (observation number 1 corresponded to the first location recorded at the capture site, just before release; number 2 was the location recorded 30 minutes later, and so on). For the others predictor variables, we considered the tracking day (tracking day 1 corresponded to the day of tagging, tracking day 2 to the second day of tracking, and so on) and calculated the average daily temperature from the outdoor temperatures recorded throughout the same day. Finally, we constructed a "time of day" variable. Day and night lengths vary with every day in the same geographical area. We used R's "maptools" package to obtain the time of the beginning of dawn, sunrise, sunset and the end of dusk for each monitoring day. We adopted the civil definition of the beginning of dawn and the end of dusk, i.e. the moment when the centre of the sun is 6° below the horizon in the

morning or evening. We then associated one of the four phases of the day (dawn, day, dusk, night) with each record. Considering the handling conditions of the 10 individuals to be similar (time of day, restraint technique, and handling duration), we did not include this factor in subsequent analyses. As no juveniles were fitted and the distinction between adults and sub-adults was made on the basis of morphological criteria, we did not use age class as a predictor variable.

We built several models for each analysis; starting from the most complete. Then, we removed non-significant variables until obtaining a model with only significant ones. We selected final models based on a comparison of residual deviances using an ANOVA test and their Akaike Information Criterion (AIC). When several models showed similar performance, we kept the most parsimonious one.

The main result is that wild boar's activity is reduced during the first month of tracking, with no clear pattern of the post-capture effect on movement speed (**Figures 1a & 1b**). On the other hand, outdoor temperature had a significant influence on movement speed (**Figure 1c**), which could be at least partially explained by the time of day the temperatures were recorded, with the coolest temperatures being recorded during night-time activity periods (**Table 2; Figure 1d**). Indeed, wild boar are known to be nocturnal animals, especially when evolving in anthropized areas (Ohashi et al. 2013; Rosalino et al. 2022). However, predictions of DDT and DAR from the best models confirm the species' sensitivity to heat: 1/ DDT decreased above an average temperature of 15°C recorded over 24 hours (**Figure 1e**) and was significantly higher in winter than in autumn and summer (as was speed of movements) (**Table 2; Figure 1f**) and 2/ DAR decreased when mean daily temperature exceeded about 20°C (**Figure 1g**). Finally, we found a significant difference in DDT between monitoring zones (**Table 2; Figure 1h**), and more specifically, between the area characterized by the highest human disturbance (Entre-deux-mers) and the one with the highest tranquillity (Jalles) (see Step 4 in the manuscript).

Ensing EP, Ciuti S, de Wijs FALM., Lentferink DH., ten Hoedt A et al (2014) GPS based daily activity patterns in European red deer and North American elk (Cervus elaphus): indication for a weak circadian clock in ungulates. PLoS ONE, 9(9):1-11

Gentsch RP, Kjellander P, Röken BO (2018) Cortisol response of wild ungulates to trauma situations: hunting is not necessarily the worst stressor. Eur J Wildl Res 64:1- 12

Koch CE, Leinweber B, Drengberg BC, Blaum C, Oster H (2017) Interaction between circadian rhythms and stress. Neurobiol Stress 6:57-67

Miettinen E, Melin M, Holmala K, Meller A, Väänänen V-M et al (2023) Home ranges and movement patterns of wild boars (Sus scrofa) at the northern edge of the species' distribution range. Mamm Res 68:611–623

Ohashi H, Saito M, Horie R, Tsunoda H et al (2013) Differences in the activity pattern of the wild boar *Sus scrofa* related to human disturbance. Eur J Wildl Res 59:167–177

Rosalino LM, Teixeira D, Camarinha C, Pereira G, Magalhães A, et al (2022) Even generalist and resilient species are affected by anthropic disturbance: Evidence from wild boar activity patterns in a Mediterranean landscape. Mamm Res 67(3):317–325

Thurfjell H, Spong G, Ericsson G (2014) Effects of weather, season, and daylight on female wild boar movement. Acta Theriol 59:467–472

Ulrich-Lai Y, Herman J (2009) Neural regulation of endocrine and autonomic stress responses. Nat Rev Neurosci 10:397–409

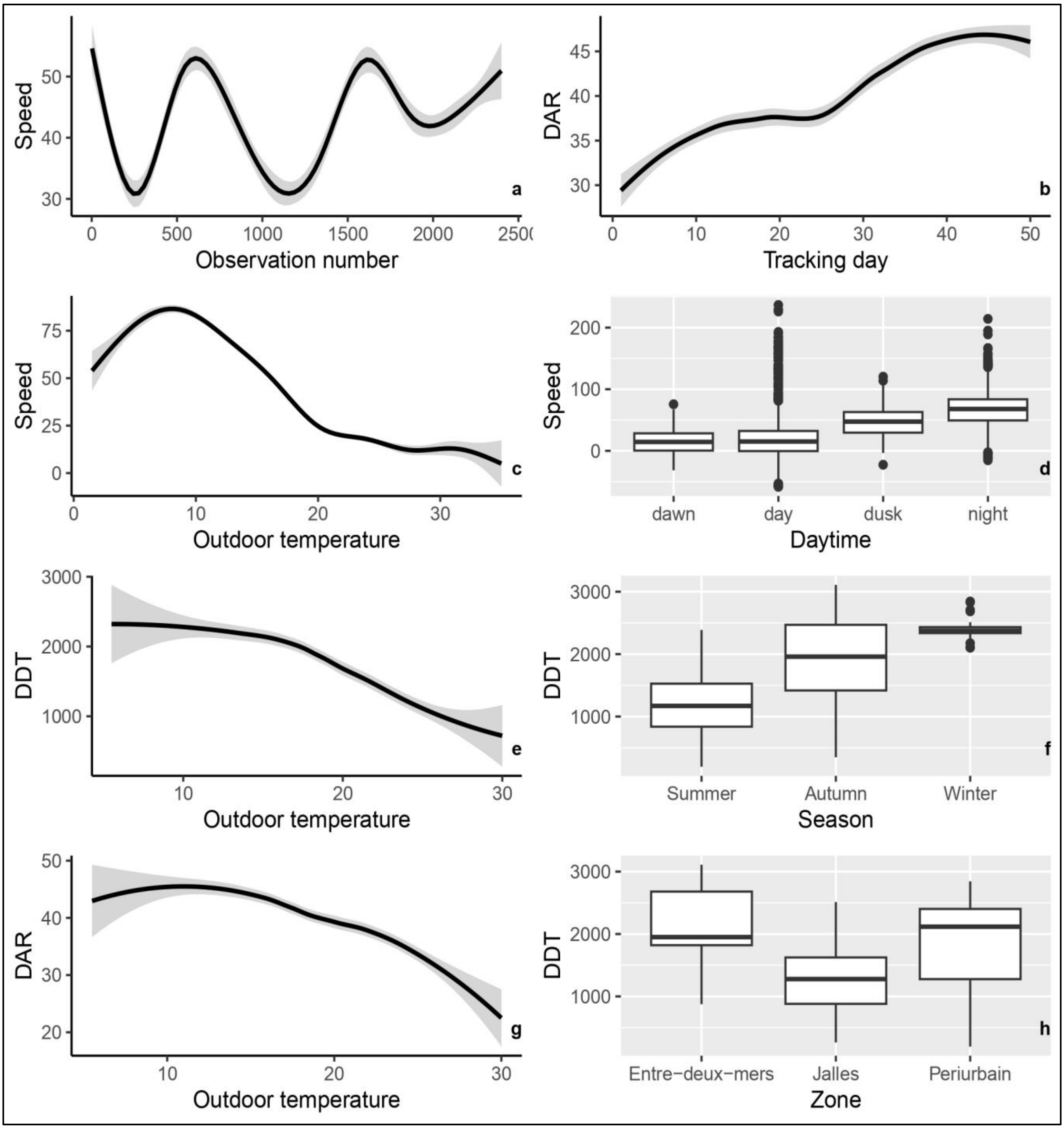

Figure 1 Values of Speed of movements, DDT and DAR during the first 50 days of monitoring, predicted by the best GAM: a) and b) Effect of the post-capture delay on Speed and on DAR, respectively; c), e) and g) Effect of the outdoor temperature on Speed, DDT and DAR, respectively; d) Effect of the phase of the day on Speed; f) Effect of the season on DDT; h) Effect of the monitoring zone on DDT. Estimated standard errors are represented by the grey-shaded areas

Table 1 Final GAMMs for predicting Speed, DDT and DAR of urban wild boar during the first 50 days of monitoring and their characteristics ((-): Not included in final model)

	Final GAMM for Speed			Final GAMM for DDT			Final GAMM for DAR		
	Df	F-value	p-value	Df	F-value	p-value	Df	F-value	p-value
Observation number	45.21	7.70	$<2.2e^{-16}$						
Tracking day				(-)	(-)	(-)	4.68	4.39	0.00034
Zone	2	1.33	0.26	2	2.93	0.055	2	0.90	0.408
Season	2	3.10	0.045	2	2.58	0.077	2	2.52	0.082
Sex	1	1.49	0.22	1	0.034	0.855	(-)	(-)	(-)
Time of day	3	92.96	$<2.2e^{-16}$						
Temperature	6.09	59.55	$<2.2e^{-16}$						
Mean daily temperature				2.50	5.80	0.0005	3.17	5.74	0.00016
Individual	5.71	37.52	$<2.2e^{-16}$	5.02	8.79	$<2.2e^{-16}$	4.40	2.65	0.00033
R2(adj)			0.137			0.269			0.157

Table 2 Parametric coefficients of categorical variables included in final GAMMs for predicting Speed, DDT and DAR of urban wild boar during the first 50 days of monitoring

	Final GAMM for Speed				Final GAMM for DDT				Final GAMM for DAR			
	Estimate	Std Error	t-value	p-value	Estimate	Std Error	t-value	p-value	Estimate	Std Error	t-value	p-value
Zone (Jalles as ref mode)												
Entre-deux-mers	28.63	19.22	1.49	0.136	1126.31	468.13	2.41	0.017	5.77	4.39	1.31	0.189
Periurban	27.63	21.46	1.29	0.198	744.34	529.65	1.41	0.161	3.67	4.35	0.84	0.399
Season (Winter as ref mode)												
Autumn	-14.51	6.38	-2.28	0.023	-703.73	345.82	-2.04	0.042	3.50	4.89	0.72	0.475
Summer	-25.34	11.56	-2.19	0.029	-1084.70	502.81	-2.16	0.031	12.15	6.22	1.95	0.052
Time of day (night as ref mode)												
Dawn	-47.25	6.00	-7.87	$3.76e^{-15}$								
Day	-33.10	2.13	-15.53	$<2.2e^{-16}$								
Dusk	-5.09	6.52	-0.78	0.435								
Sex (Female as ref mode)												
Male	-24.69	20.24	-1.22	0.223	-95.25	519.75	-0.18	0.855				
